# Dispelling Vaccine Legal Myths

**DOI:** 10.1017/jme.2025.10203

**Published:** 2026

**Authors:** James G. Hodge

**Affiliations:** Sandra Day O’Connor College of Law, Arizona State University, United States

**Keywords:** vaccine, law, myths, exceptions, constitution

## Abstract

Major shifts underway in US vaccine policies reflect widespread misinformation, notably including unproven claims of harms from vaccines. Vaccination misconceptions also include an array of falsities about the scope and extent of governmental powers and protections. Exposing these “legal myths” clarifies existing foundations of vaccine laws and policies, providing guidance on appropriate responses to quell vaccine hesitancy.

Perhaps never before in modern US history have vaccine-related rumors, misinformation, and myths been more prevalent or damaging.^1^ Falsely linking childhood vaccines in some way to growing numbers of diagnosed cases of autism, for example, is not new.^2^ Alleging that such vaccines are a definitive cause of autism is, especially against a paucity of any new actual evidence supporting such claims.^3^ While ties between autism and vaccines remain at the forefront of vaccine myths, misinformation extends far beyond this debunked premise. Over the past few years, vaccines have allegedly been associated with manifold additional harms, including arthritis,^4^ attention-deficit/hyperactivity disorder,^5^ diabetes,^6^ infertility,^7^ multiple sclerosis,^8^ narcolepsy,^9^ and venous thromboembolism.^10^ In October 2025, public and private sources queried whether vaccines actually poison people through trace additives like aluminum designed to make active immunologic agents more efficient.^11^

The list of alleged vaccine-related harms goes on and on. Many purported harms resonate with what I refer to as the “flat tire” scenario. Anyone who hits a bump in the road and immediately experiences a flat tire may tend to blame the bump. Sometimes they are right. What one may not suspect, however, is that the actual cause of the flat tire is the nail on the road run over two days prior. The tire was going flat over time. The bump in the road merely provided the first clear sign of its failure. By analogy, the inception of specific illnesses or conditions within proximity to the administration of vaccines, particularly during childhood, leads many to point to vaccines as the causative agent. In so doing they look past myriad genetic, environmental, or other factors impacting a child’s development. They do not believe these are the causes of a child’s malady. It is easier to blame the bump on the road — that is, the vaccination.

The paradox of the “flat tire” scenario is that persons suspecting vaccines to be the premier cause of a child’s adverse medical condition are likely blinded to how vaccines are key to preventing the onset of other harmful illnesses like measles, mumps, rubella, and polio.^12^ People tend not to worry about medical harms they avoid, but rather the ones they actually experience. The myth that vaccines are the cause of unrelated medical problems propels anti-vaccinationists in part to reform long-standing requirements that populations be immunized.^13^

Myths are not limited to causative suspicions about vaccine-related harms. They underlie vaccine laws and policies as well. As listed and analyzed below, a series of vaccine legal myths sway public perceptions and contribute to choices and behaviors contrary to the protection and promotion of the public’s health. Just as trustworthy science and advanced health literacy can dissipate fact-based myths surrounding vaccines,^14^ improved legal and policy understanding can unravel and dispel misperceptions or fabrications about the role of law and vaccinations.

## Myth 1: Autonomous adults can be legally compelled to get vaccinated

A lot of Americans may think this is true, but it is not. Neither government nor private sector actors can physically compel autonomous adults to be vaccinated against their will or without their consent.^15^ The key distinction underlying this myth relates to the legal distinctions between compulsion and mandates.

People routinely confuse vaccine mandates with compelled vaccinations. It is understandable in part, given the unfortunate, traditional use of the term “vaccine mandate” for what is really “conditional vaccination.” Vaccine mandates do not allow government or others to compel individuals to get vaccinated. Rather, they set conditions upon societal participation in select activities (e.g., attending school, working, traveling) as an incentive to choose vaccination. Some may view these mandates as heavy-handed. Others may suggest they do not offer much of a choice at all. Florida and select other states have proposed ending all legal vaccine mandates.^16^ Idaho passed legislation in 2025 over initial gubernatorial objections, accomplishing just that.^17^

Yet vaccine mandate laws do not physically require autonomous persons to get vaccinated. Compulsory vaccinations used to be practiced in select locales in the US, especially during outbreaks of smallpox or other serious diseases.^18^ Some people were physically administered vaccines against their will.^19^ As an affront to bodily liberty, compelled vaccinations of autonomous adults are a thing of the past. Conditional vaccine laws, however, remain a viable and important public health intervention.

## Myth 2: Government cannot require children or other wards to be vaccinated

If government cannot physically compel autonomous adults to be vaccinated, such limits may logically extend to their children or other “wards” of the state. This is, however, only partially true. Public health officials, child protective service workers, and courts are loath to interfere with parental decisions concerning medical treatments, including vaccinations, for their children. Yet in rare cases when children or other wards are directly placed in harm’s way due to failures to vaccinate, government actors can require vaccinations.^20^ In some states, parents refusing to vaccinate their children may be charged with neglect, especially during disease outbreaks.^21^ However, most states disallow criminal charges for parental denials of basic medical services.^22^

Distinct vaccine requirements for children and other wards, as contrasted with autonomous adults, arise from applications of *parens patriae* powers.^23^ These powers, constitutionally reserved to states along with police powers, justify governmental interventions to assure the health and safety of a host of non-autonomous adults, including prisoners, those lacking mental capacities, persons with disabilities, and, at times, children.

## Myth 3: Religious or philosophical exemptions to vaccine mandates are constitutionally required

Most states currently allow persons to exercise religious or philosophical exemptions to many vaccine mandates. That some states like California, New York, and West Virginia do not essentially exposes this myth.^24^ If these exemptions were constitutionally protected, states would have to provide them. To date, the US Supreme Court has not found that religious or philosophical exemptions to vaccine mandates are required under the First Amendment or other grounds.^25^ Except for a select few lower court decisions to the contrary,^26^ state lawmakers choose whether to allow these exemptions.

How much longer this remains the status quo is uncertain. Despite dozens of opportunities, the Supreme Court has repeatedly refused to hear a definitive case on the constitutionality of religious or philosophical exemptions. In two 2025 companion cases in New York, however, plaintiffs have petitioned the Supreme Court to hear their challenges to denials of religious exemptions to vaccines in violation of First Amendment free exercise principles and federal civil rights legislation.^27^ Whether the Court agrees to hear either of these cases is indeterminate. The stakes are high. Predictable widespread uptake of religious exemptions to vaccine mandates, especially in some regions of the country, would inevitably lead to outbreaks of preventable childhood diseases like measles among unvaccinated populations.^28^

## Myth 4: Treatment for common, vaccine-preventable illnesses can be withheld from unvaccinated persons

Another misperception is that persons who willingly choose against vaccination, notably during public health emergencies like the COVID-19 pandemic, can be effectively de-prioritized for treatments that safe and effective vaccines could have prevented. To the extent that select decisions via medical personnel denying care solely to unvaccinated patients arose during the COVID-19 pandemic,^29^ those actions were unwarranted.

Denying persons treatment for conditions they might have avoided is precarious. Doctors are highly reticent to ration medical care based on patients’ substantive choices or behaviors. The line drawing would never end. Nearly everyone acts or makes decisions that contribute adversely to their medical conditions. Personal reasons to avoid vaccines are manifold. Some act on their own fears of vaccine-related harms or misinformation. Others invoke legally allowed religious or philosophical exemptions. Still others are immunocompromised. Millions more Americans simply lack access to or financial resources for vaccines or distrust government. Which of these reasons to avoid vaccinations alone or in combination justifies the rescission of needed medical attention? None. Denying care based on judgment calls is legally discriminatory and ethically unjustifiable. Besides, the price one pays for rejecting vaccines — namely, the onset of preventable, sometimes lethal diseases — is high enough.

## Myth 5: Pediatricians cannot discriminate against parents for their refusal to vaccinate their children

If treatments for vaccine-preventable conditions cannot be withheld solely related to one’s choice to get vaccinated, surely pediatricians cannot refuse to take on kids whose parents do not want them vaccinated to begin with.^30^ Actually, pediatricians can refuse to treat such patients and regularly do. Doctors have every right to make conscious choices in the interests of their patients and those around them (e.g., in pediatric waiting rooms) to reject unvaccinated kids as new patients.^31^

How is it not legally discriminatory to turn away unvaccinated kids, especially since they may need pediatric care the most? It is a matter of timing. A pediatrician refusing to take on unvaccinated kids as patients has not established a legally recognized relationship with either the kids or their parents. Absent such a relationship, there is no duty to treat.^32^ Conversely, once a doctor has established such a relationship with kids, or any other patients for that matter, their continued obligation to treat them for preventable conditions (including those which vaccinations may have avoided) may follow as a matter of law.^33^

## Myth 6: Vaccines available to patients are legally approved by FDA

While most vaccines administered nationally are fully approved by FDA for their intended purposes, there are exceptions. Initial COVID-19 vaccinations, for example, were legally administered to millions of Americans without formal FDA approval. As noted above, the legal distinction here is a term of art. FDA has the capacity to fully approve vaccines, which is standard for most common inoculations.^34^ Yet the agency can also issue emergency use authorizations (EUAs) for vaccines produced in response to emerging infectious conditions like COVID-19.^35^ These vaccines are legally “authorized,” but not “approved.” Full approvals may follow months or years later, as they have for multiple COVID-19 vaccines;^36^ however, the lack thereof does not mean vaccines cannot be lawfully administered. While initial EUAs or full approvals each provide their own distinct legal route to accessing vaccines, FDA’s potential to reverse or deny EUAs for future emerging vaccines (e.g., H5N1 — avian flu)^37^ or withdraw approvals of long-standing vaccines like polio^38^ presents substantial threats to the public’s health.

## Myth 7: Only doctors can lawfully prescribe vaccinations

Like many medications, vaccines may require prescriptions prior to their administration,^39^ which doctors are authorized to issue, even off-label.^40^ Thus doctors can prescribe specific vaccines for patients (e.g., those with immunocompromised statuses) even if FDA does not fully approve or authorize them for such persons. The notion that only doctors can prescribe vaccines is a myth, however. Doctors can effectively transfer or bestow their prescription authorities to other licensed health care workers (HCWs), like registered nurses and pharmacists.^41^ Acting pursuant to standing orders or other legal authorizations under physician supervision, HCWs can administer vaccines just the same as doctors. In some states, licensed HCWs are directly allowed to prescribe and administer vaccines to patients.^42^

## Myth 8: States are legally required to follow CDC vaccine guidelines

This falsehood has arisen most recently in relation to national backlash against misguidance from CDC’s reconstituted Advisory Committee on Immunization Practices (ACIP).^43^ States’ concerns are palpable. For decades, multiple states have essentially lassoed their vaccination recommendations to ACIP as a trusted, reliable, and scientific source for sound vaccine policies.^44^ If ACIP irresponsibly drops its longstanding recommendations for a variety of childhood and adult vaccines, states may still have to follow such ill-advised, inappropriate rescissions. Blind adherence by law to unsubstantiated ACIP guidance may diminish longstanding vaccine rates among children or other populations.

States have every legal opportunity to untether themselves from CDC guidance. Reliance on ACIP recommendations is not controlled by the federal government.^45^ State-based regulatory or legislative fixes can obviate state vaccination requirements and ACIP recommendations. Multiple Pacific coast and Northeast corridor states and localities are already substituting their own scientifically-grounded vaccination recommendations for those emanating from ACIP.^46^ Governors and other leaders elsewhere are likely to follow.

## Myth 9: Manufacturers are not liable to consumers for harms directly tied to vaccinations

People believe vaccine manufacturers avoid substantial liability through pre-ordained protections crafted by Congress as an incentive to produce vaccines. It is true that manufacturers are insulated to a degree from liability, but not completely. Where potential injuries due to vaccine administration are alleged, patients (or their families) can recover direct costs through federally funded programs like the national Vaccine Injury Compensation Program (VICP).^47^ Since 1986, this program has provided a direct path for injured persons to obtain recourse. Additional vaccine injury compensation programs arise in other contexts (e.g., PREP Act emergency declarations).^48^

Successful injury claims are few. Administration of an overwhelming majority of vaccines leads to no direct harms to recipients. According to the Health Resources and Services Administration (HRSA), citing CDC data, among the over 5 billion covered vaccines administered in the US between 2006 and 2023, just over 10,000 injury claims through the VICP were compensated. “This means for every 1 million doses of vaccine that were distributed,” concludes HRSA, “approximately 1 individual was compensated.”^49^

Liability protections under these Congressionally authorized programs depend on continued federal support, buttressed in part by excise taxes imposed on covered vaccinations.^50^ To date, thousands of Americans have been paid directly through VICP.^51^ Those denied support can always bring a separate case in court.^52^ Legally proving a vaccine caused injury is not easy in part because vaccines are so safe, but the idea that manufacturers are not liable for their products is more myth than reality.

## Myth 10: Vaccine misinformation is constitutionally protected speech

The idea that Americans have every right to spread known misinformation about vaccinations is sensational, especially because in many cases, they do not. There is no right to share misinformation about specific products, companies, or practices via private sector social platforms or other non-governmental venues. These platforms can effectively shut down vaccine misinformation messaging. First Amendment freedoms are not implicated so long as government is not directly involved in policing such restraints. And while government may have little recourse to stymie social media banter over vaccines, when misinformation ventures into the realm of commercial speech, government can effectively shut it down. There is no First Amendment right, for example, to knowingly share falsehoods or other misinformation about vaccines for commercial purposes.^53^ It can also be legally treacherous. Individuals who share false information about vaccinations may face criminal or civil charges of libel, defamation, or fraud.^54^

Deciphering the legal landscape surrounding vaccines in the United States is challenged by legal misinformation, just as material and factual misstatements regarding vaccinations contribute to the “flat tire” scenario more broadly. Key takeaways from the exposure of these legal myths guide laws and policies ahead. Foremost, appropriate vaccination laws do not physically compel autonomous persons to be inoculated. Only rarely are nonautonomous persons like children or other “wards” vaccinated outside their or their caregivers’ consent. Otherwise, public health laws set reasonable, constitutionally sound conditions (with some exceptions) for people to choose vaccinations in the interests of their and the community’s health. People who avoid vaccinations for religious, philosophical, or other reasons may legally be denied access to key services and benefits, including initial doctor-patient relationships, but not treatment for their vaccine-preventable conditions.

Safe and effective vaccines authorized or approved by FDA may be prescribed and administered by a range of state-based licensed health care providers based on recommendations issued by ACIP and other entities legally recognized by states seeking to assure access to vaccines. In rare cases where vaccines may actually harm recipients, manufacturers and government share prospective liability for such injuries. Americans’ freedom of speech to share vaccine-related information is extensive, but does not include spreading known misinformation, or defaming specific products or manufacturers through commercial sources.

Ultimately, dispelling vaccine legal myths is profoundly challenged by rampant misinformation, governmental distrust, and rejection of scientific bases. Law- and policy-makers facing anti-vaccinationist views should take stock in over a century of pro-vaccination laws. Reasonable laws encouraging widespread vaccination of populations are not only highly effective in preventing morbidity and saving lives, they enhance health care services, facilitate education, enable safe workplaces, contribute to improved economic outcomes, and heighten societal stability.
